# Is prevention of systemic lupus erythematosus a goal?

**DOI:** 10.1186/ar4618

**Published:** 2014-09-18

**Authors:** Nancy J Olsen, David R Karp

**Affiliations:** 1 Penn State MS Hershey Medical Center, Hershey, PA, USA; 2 University of Texas Southwestern Medical Center, Dallas, TX, USA

## Background

Prevention of systemic lupus erythematosus (SLE) presents many challenges. By contrast, prevention of acquired immunodeficiency syndrome (AIDS) is relatively straightforward: an infective agent has been identified, risk behaviors are well-delineated, antiviral therapeutics are highly effective and neonates have been apparently cured. Lupus is a more complex disease, with a significant but incompletely defined genetic component, widely heterogeneous manifestations and major gaps in knowledge about pathogenesis.

## Methods

The characteristic features of SLE can be exploited in the quest for preventive strategies. One of these is the presence of a latent phase during which expressed autoantibodies are increasing in number and complexity prior to the onset of clinical symptoms. This offers a path to the development of screening blood tests that would be cost-effective and generally acceptable to subjects. ANA alone is clearly not sufficient to establish risk, as it is highly prevalent in the healthy population. Alternatively, a panel of autoantibodies, possibly combined with cytokines and gene expression levels, might be useful. The skewed demographics of SLE can also be exploited, including the higher prevalence in females, first-degree relatives and individuals < 40 years old, permitting focus on those who are most likely to be at risk.

## Results

A composite index, with demographics and multiplex blood autoantibody profiles, has been proposed. This index showed statistically significant correlation with progression of disease in a small prospective cohort (*P *= 1 × 10^-7^). As the science improves, the risk definition could be augmented with targeted genetic information, as is now available for several inherited cancers; even the simple inclusion of family history as a proxy for genetic input might be of value. All such screening efforts would be for naught in the absence of an available intervention. Fortunately, several candidate treatments are available for the incomplete lupus phenotypes, and precedent for using therapeutics in individuals who are not yet ill has been established in other conditions, notably type I diabetes mellitus.

## Conclusions

It is timely to propose a prevention trial in individuals at high risk for development of SLE. A placebo arm would probably be required in such a study to show that the enrollment criteria successfully identified high-risk individuals. Challenges to trial design include acceptance of "no treatment" by persons profiled as high risk and the relatively long timeline likely to be required to achieve observable clinical change. However, despite these issues, available tools and therapeutics make prevention trials in SLE a feasible, near-term prospect.

